# LMIC Research Centers’ Experiences Hosting U.S. and LMIC Trainees: Evaluation of the Fogarty Global Health Fellows and Scholars Program, 2012 to 2020

**DOI:** 10.4269/ajtmh.22-0595

**Published:** 2023-02-20

**Authors:** Baba M. Musa, Leslie Pierce, Leslie C. M. Johnson, Liping Du, Faisal S. Dankishiya, M. Shannon Byers, Donna J. Ingles, Douglas C. Heimburger, Muktar H. Aliyu

**Affiliations:** ^1^ Department of Medicine, Aminu Kano Teaching Hospital, Bayero University, Kano, Nigeria;; ^2^ Vanderbilt Institute for Global Health, Vanderbilt University Medical Center, Nashville, Tennessee;; ^3^ Department of Family and Preventive Medicine, School of Medicine, Emory University, Atlanta, Georgia;; ^4^ Department of Biostatistics, Vanderbilt University Medical Center, Nashville, Tennessee;; ^5^ Aminu Kano Teaching Hospital, Kano, Nigeria;; ^6^ Office of the Vice Provost for Research and Innovation, Vanderbilt University, Nashville, Tennessee

## Abstract

In this mixed-methods study, we explore themes that emerged from a survey assessing the programmatic experiences of mentors and administrators at institutions in low- and middle-income countries (LMICs) hosting trainees supported by the Fogarty International Center’s Global Health Program for Fellows and Scholars. A total of 89 of 170 potential respondents representing 31 countries completed the survey (response rate, 52.4%). There was agreement among respondents that their institutions received sufficient funds to support trainees and had the capacity to manage operational and financial aspects of the program. A majority also agreed that both LMIC and U.S. trainees were beneficial to the host institutions, and that trainee projects were relevant to the needs of the host country. Respondents felt that program benefits to LMIC trainees could be improved by increasing the research consumables budget, increasing the flexibility of program timelines, and increasing engagement between LMIC and U.S. trainees and institutions. Respondents indicated that both U.S. and LMIC trainees behaved professionally (including demonstrating respectful and ethical behavior) and took appropriate initiative to conduct their research projects. Findings from this study will help inform innovations to similar training initiatives that will enhance sustainability and improve program performance, and will be responsive to local needs.

## INTRODUCTION

Increasing interactions between the global North and South have enhanced interest in global health.^1^ Programs leveraging this paradigm and those aimed at optimizing and enhancing the productivity of these interactions^2^ have led to improved capacity among trainees, especially in research skills, and have facilitated a closer look at best practices in global health interactions.^3^ In 2003, the Fogarty International Center (FIC) of the U.S. NIH, with additional support from other NIH institutes and centers and the Ellison Medical Foundation, established a program to provide training opportunities for U.S. professional and doctoral students in global health research. In 2008, the program was expanded in size and scope by adding postdoctoral clinical and research fellows (both from the United States and low- and middle-income countries [LMICs]), and was renamed the Fogarty International Clinical Research Scholars and Fellows Program, which was administered by a grant to Vanderbilt University as the Administrative Support Center. In 2012, it was reorganized as five consortia of U.S. institutions, and again renamed to the Fogarty Global Health Program for Fellows and Scholars (FGHFS).^4^ In 2017, a sixth consortium was added. Each consortium comprised four U.S. institutions with at least six other collaborating universities in LMICs.^5^ Throughout all iterations of the program, it has remained a 1-year mentored research training experience anchored at collaborating LMIC institutions, some of which have participated in the program since 2003.^6^^,^^7^

Global health research capacity development is reinforced when embedded within the framework of mutually respectful institutional partnerships. When such partnerships are optimized, they create a robust system of collaboration, shared knowledge of local perspectives, and vision anchored on common objectives and outcome benchmarks.^8^^,^^9^ In this context, it is important for regional voices to be heard and for the needs of local mentors to be acknowledged and prioritized, enabling increased visibility of domestic mentors in decision making, including selection of eligible trainees. In this article, the FGHFS consortia explore a spectrum of themes that emerged from a survey assessing the programmatic experiences of mentors and administrators at LMIC host institutions: the participation intent by LMIC host mentors in areas of decision making, including selection of potential mentees; the capacity to manage grants by LMIC institutions; the impact of U.S. and LMIC trainees on LMIC host sites and institutions; the relevance of trainee projects to the needs of host institutions; the expectations of trainees at the commencement of training; and the adaptation of U.S. trainees to cultural sensitivities and their professional conduct at the LMIC host sites.

## MATERIALS AND METHODS

### Setting and participants.

Between 2012 and 2021, the FGHFS program supported 892 trainees across 40 countries. At the time of the study, 22 LMIC sites were actively accepting trainees for participation in the program. Low- and middle-income country sites that had hosted at least one trainee for a full training period (1 year) between 2012 and 2021 were eligible for participation. To ensure adequate feedback from both the administrative and mentor perspectives, at least one scientific mentor and one administrator were identified from each site. In some cases, the same person filled both roles, so only one survey response was received from these sites.

### Objective.

The purpose of this study was 1) to capture the perspectives of mentors and administrators at the LMIC host institutions and project sites on the burdens and benefits of participating in these training programs from 2012 through 2021, 2) to determine ways in which the partnerships between LMIC and U.S. lead sites could be improved, and 3) to assess trainee preparedness and conduct.

### Survey development.

We reviewed published literature examining the benefits and burdens on institutions and preceptors in LMIC settings that hosted international clinical or research-focused trainees. This review revealed six important themes: 1) administrative responsibilities and site capacity; 2) site engagement in the selection of trainees; 3) general impact on the host institution; 4) trainee attitudes, behavior, and professionalism; 5) trainee preparation and knowledge; and 6) feedback mechanisms.^10^^–^^16^ We constructed a 175-item survey that touched on each of these content areas. Because the program included both U.S. and LMIC trainees, we designated separate responses for each citizenship category and included branched-logic distinctions where applicable. Demographic information on the locations of the host institutions and experiences of the survey respondents with the program were also included. The format included Likert-scale, multiple-choice, and open-ended free-text questions.

### Data collection.

Program managers from the six active FGHFS consortia (as of 2020) provided lists of administrative and scientific mentor contacts for each of their LMIC sites. The survey was disseminated via e-mail, with reminders for nonresponders, and responses were collected in REDCap, a secure, Web-based data collection and database platform.^17^

### Quantitative data analysis.

One respondent (of 89 total respondents) took the survey twice, corresponding to roles with two distinct FGHFS consortia. The first response of this respondent was excluded from all analyses. All available data from the 88 surveys (each from a unique respondent) were included for analysis, although five surveys were less than 75% complete. The overall data, or data for a group (such as those grouped based on the roles of respondents, or hosting U.S. or LMIC trainees or both), were summarized using descriptive statistics such as means ± SD for continuous variables, and frequencies and percentages for categorical variables. All analyses and graphs were completed using software R.4.1.3 (R Foundation for Statistical Computing, Vienna, Austria), and R packages such as ‘Hmisc’^18^ and ‘ggplot2.’^19^

### Qualitative data analysis.

All free-text responses were extracted, deidentified, compiled into a spreadsheet, and organized according to the corresponding survey question. Data abstraction and interpretation were completed using a qualitative content analysis approach.^20^ Two reviewers open-coded the text data independently, a process during which discrete data segments are assigned codes that describe or classify the phenomenon represented in the data.^21^ A consensus was reached on code names and application, then data across respondents were compared to group codes into categories based on similarities. Themes across the categories were identified in relation to priorities around program engagement and timelines, and in relation to approaches for supporting inclusive program development and growth that brings visibility to the trainees and host institutions. Data were then compared across respondents based on the number of trainees their institution hosted to identify differences in how host sites experienced participation in the FGHFS program, and ways the program benefited their institutions and supported trainees’ research and career advancement (see Supplemental Table 1 for results of this subgroup comparison). For both quantitative and qualitative data, we anonymized responses to protect respondent confidentiality.

## RESULTS

### Respondent demographics and experience with program and trainees.

A total of 89 of 170 potential respondents representing 31 countries took the survey (response rate, 52.4%). Forty-three percent of respondents (*n* = 38) were female (Table 1). Half of the respondents were from India (*n* = 11, 12%), Zambia (*n* = 7, 8%), Peru (*n* = 6, 7%), South Africa (*n* = 6, 7%), Tanzania (*n* = 6, 7%), and Uganda (*n* = 6, 7%) collectively. The majority of respondents (*n* = 71, 81%) described their roles in relation to the program as including oversight, supervision, or teaching (also called the mentor role). Respondents reported an average of 7.7 ± 5.5 years (SD) of experience with the program. More than half the respondents reported hosting both U.S. and LMIC trainees (*n* = 49, 56%). Two thirds of respondents with a mentor role reported spending between 5 and 20 contact hours per month with each trainee (*n* = 47, 66%). Most respondent institutions had hosted fewer than five trainees at their sites (*n* = 51, 58%) and indicated being at below capacity (i.e., could support more trainees; *n* = 67, 77%; Table 1). The length of the 1-year training was perceived as optimal by most respondents with a mentor role (*n* = 56, 80%).

**Table 1:** Characteristics of respondents and host sites

Characteristic	*n*	%
Gender (*N* = 88)		
Female	38	43.2
Male	50	56.8
Consortium (*N* = 88)		
GHES	16	18.2
GloCal	16	18.2
HBNU	9	10.2
NPGH	11	12.5
UJMT	9	10.2
VECD	22	25.0
> 1 consortium	5	5.7
Role in program (*N* = 87)		
Administrative management	16	18.4
Oversight, supervision, teaching	63	72.4
Both of the above	8	9.2
Trainee-type hosted (*N* = 88)		
LMIC	18	20.5
U.S.	21	23.9
Both of the above	49	55.7
Experience with the program, years; mean ± SD (*N* = 86)	7.7 ± 5.5	
No. of fellows hosted (*N* = 88)		
< 5	51	58.0
5–10	22	25.0
11–15	8	9.1
16–20	5	5.7
> 20	2	2.3
Capacity to host trainees (*N* = 87)		
At capacity	20	23.0
Could host more	67	77.0
Hours/month spent with trainee (*N* = 71, mentors only)		
0–5	20	28.2
6–10	27	38.0
11–20	20	28.2
> 20	4	5.6
Program length (*N* = 70, mentors only)		
Just right	56	80.0
Too short	14	20.0

GHES = Global Health Equity Scholars; GloCal = University of California Global Health Institute Program for Fellows and Scholars; HBNU = Harvard–Boston–Northwestern–University of New Mexico consortium; LMIC = low- and middle-income country; NPGH = Northern Pacific Global Health Fellows Program; UJMT = UJMT Fogarty Global Health Fellows Consortium; U.S. = United States; VECD = Vanderbilt–Emory–Cornell–Duke Consortium for Global Health Fellows.

### Site support, communication with consortia, and feedback from trainees.

Of the 23 administrators who responded to the survey, more than half (*n* = 14, 61%) agreed or strongly agreed that their institution received enough funds to support the trainees (Table 2). Respondents advocated for additional funding to secure protected research time for trainees and their mentors, and noted that expanded funding could allow for a larger program capacity (see Table 3 for an expanded description of the open-ended survey responses). They also agreed or strongly agreed that their research institutions had the capacity to manage the subcontracts and funding associated with the program (*n* = 22, 96%; Table 2). Most host institution administrators were comfortable reporting problems or issues to a consortium/program contact (agree/strongly agree: *n* = 20, 87%). A similar proportion of administrators rated the amount of communication between the host institution and the consortia/program as “appropriate.” However, only 22% of respondents (*n* = 5) with administrative roles reported being involved in application review and trainee selection (data not shown).

**Table 2:** Consortium support, site capacity, and communication with sites (respondents with administrative roles only, *N* = 23)

Variable	*n*	%
Institution is provided with enough funds to host the trainees		
Strongly disagree	2	8.7
Disagree	1	4.3
Neutral	6	26.1
Agree	11	47.8
Strongly agree	3	13.0
Institution has the capacity to manage subcontracts and funding		
Strongly disagree	1	4.3
Disagree	0	0.0
Neutral	0	0.0
Agree	6	26.1
Strongly agree	16	69.6
Amount of communication between host institution and consortia/program is appropriate		
Strongly disagree	0	0.0
Disagree	1	4.3
Neutral	2	8.7
Agree	10	43.5
Strongly agree	10	43.5
Comfortable reporting problems/issues to consortia/program contact		
Strongly disagree	0	0.0
Disagree	0	0.0
Neutral	3	13.0
Agree	8	34.8
Strongly agree	12	52.2

**Table 3:** Qualitative themes mapped to key quantitative outcomes

Quantitative outcomes	Qualitative themes		
	Enhancing local program ownership and LMIC trainee experiences	Prioritizing preparation for host sites and U.S. trainees	Future directions
Respondents *strongly agreed* that their institution received enough funds to support trainees, and *agreed* that their research institutions have the capacity to manage the subcontracts and funding associated with the program.	Because early and continued engagement with mentors was viewed as a central part of the program, the majority of respondents advocated for additional funding so that they could provide salary coverage for program mentors and administrators. One administrator shared that their institution is reliant on other grant funding to cover supervisors’ time when mentoring trainees whose projects are nested within ongoing research. Limited trainee research budgets can also be problematic for biomedical research projects, which often require more supplies, and meeting those needs “draws money from other grants” (no. 32).	Of those individuals who offered additional comments on what strategies are used to ensure that LMIC trainees have protected time to work on their research projects, the most common strategy was getting buy-in from institutional leadership to allow a flexible work schedule or to provide additional funding that guaranteed protected research time. These types of negotiations typically take place early in the program, when the trainees must sign institutional agreements with their employers to ensure there is a mutual understanding of how the trainee’s time will be split between their institutional responsibilities and the fellowship.	Although increasing program funding would address immediate resource gaps for some institutions, it could also allow for increased program capacity. Program growth was identified as a goal that could be achieved through increasing the number of mentors and trainees or by expanding program deliverables.
The majority of respondents *agreed or strongly agreed* that both LMIC and U.S. trainees are beneficial to the host institutions, and *agreed* that LMIC and U.S. trainee projects are relevant to the needs of the host country.	Although the respondents described previous trainees’ projects as relevant to their host countries, they all felt it important to have the trainees consult with local mentors to develop their proposals. This form of engagement was perceived as a way for the host institution to gain better insight into the potential institutional burden associated with hosting incoming trainees, based on the perceived skill levels of applicants, the types of projects they wish to pursue, and how complementary those projects are with ongoing research activities within the host institutions. Many respondents also thought that pairing U.S. and LMIC trainees could improve research productivity and collaborations between U.S. and host institutions.	N/A	The suggestions that program supervisors and administrators provided as ways to enhance the benefits of participating in the program as a host institution focused primarily on the potential for long-term program impacts, such as supporting more trainees and expanding research training objectives for trainees.
The majority of respondents *agreed* that the program benefits LMIC trainees.	Respondents felt that program benefits to LMIC trainees could be improved by increasing the research consumables budget, increasing the flexibility of program timelines, and increasing engagement between LMIC and U.S. trainees and institutions. The most frequent suggestion was that LMIC trainees be offered opportunities for experiential work in a U.S. mentor’s laboratory or facility to gain exposure to different research settings, followed by several suggestions that trainees be offered program extensions to “allow more time to ensure scientific productivity so that [trainees] have more time to achieve their career development goals” (no. 11).	Capacity building was seen as a necessary component for not only trainee advancement, but also for development of a cadre of qualified mentors prior to program commencement. Having qualified mentors is important for the success of all program trainees.	Some respondents argued for broader program eligibility criteria, particularly for LMIC trainees. Respondents indicated that having an open mind and being motivated to learn new research skills, particularly in a new discipline, proved to be a better indicator of trainee success than their education background.

LMIC = low- and middle-income country; N/A = not applicable; U.S. = United States.

**Figure 1.**
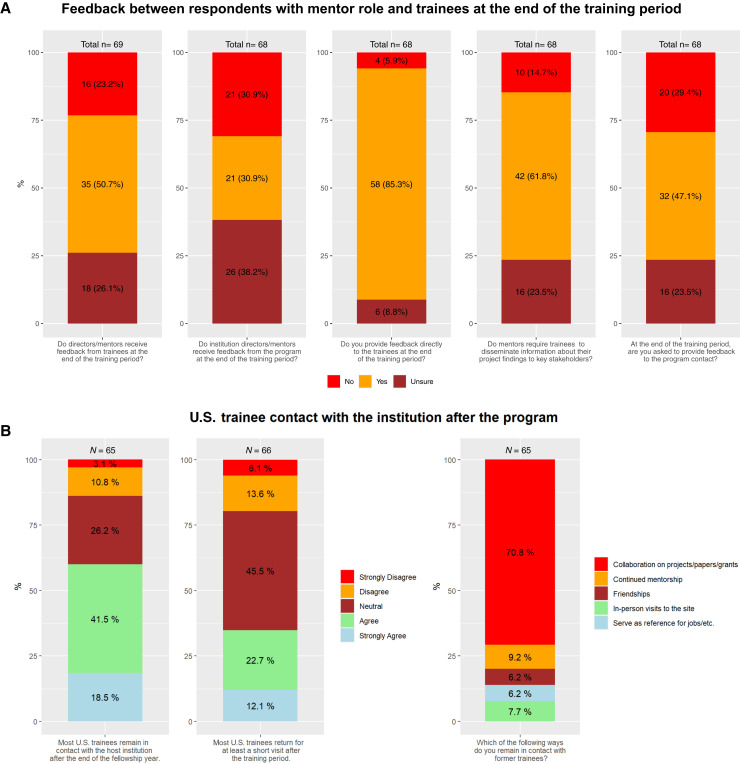
(**A**) Feedback between respondents with mentor role and trainees at the end of the training period. (**B**) U.S. trainee contact with host institution after completion of training for institutions hosting U.S. trainees.

Responses regarding feedback at the end of the training period were obtained from respondents with a mentor role, a majority of whom (*n* = 58 of 69, 85%) reported providing direct feedback to the trainees at the end of the training period (Figure 1A). In most instances (*n* = 42 of 68, 62%), mentors indicated that trainees were required to disseminate information about their project findings to key local stakeholders. Only 32 LMIC mentors (47%) reported being asked to provide feedback about the trainees to the U.S.-based program contacts at the end of the training period. Responses regarding keeping contact with U.S. trainees after the training period were obtained from 66 respondents. More than half of these respondents agreed or strongly agreed that most U.S. trainees remained in contact with the host institution after the end of the fellowship year (*n* = 39, 60%). The majority of respondents indicated collaboration on projects, papers, or grants (*n* = 46, 70.8%) as the most common way to remain in contact (Figure 1B). Several respondents also noted that many U.S. trainees returned for at least a short visit after the training period (*n* = 23 of 66, 34.8%).

### Trainee impact, professionalism, desired behavior, and knowledge.

Respondents were asked to reflect on their experiences with and impressions of the trainees with whom they had worked. The majority of respondents agreed or strongly agreed that both U.S. and LMIC trainees in the program are beneficial to the host institutions (91% and 87%, respectively; Table 4). There was also agreement regarding the benefit of the program to LMIC trainees (88%) and the relevance of U.S. and LMIC trainee projects to the needs of the host country (84% and 88%, respectively). However, respondents felt the program could be improved to enhance the experience for LMIC trainees, primarily through structuring additional engagement between U.S. and LMIC trainees and with the U.S. consortia institutions (see Table 3 for additional details). There was also agreement among respondents that U.S. and LMIC trainees did not create a burden on the host institution (76% for both groups). Survey respondents with a mentor role believed that both U.S. and LMIC trainees were adequately prepared at the beginning of their training year (98% and 95%, respectively; Table 4), and had a clear understanding of their expectations at the start of the fellowship (86% versus 79%, respectively). A majority of respondents agreed with the statement that host institutions are provided with a clear description of the trainees’ level of training (71% for both U.S. and LMIC trainees).

**Table 4:** Program benefits, relevance, trainee expectations, and level of trainee preparedness

Variable	U.S. trainees		LMIC trainees	
	*n*	%	*n*	%
Trainees in the program are beneficial to the host institution (U.S., *N* = 67; LMIC, *N* = 67)				
Strongly disagree	4	6.0	4	6.0
Disagree	0	0.0	1	1.5
Neutral	2	3.0	4	6.0
Agree	26	38.8	18	26.9
Strongly agree	35	52.2	40	59.7
Participation in the program is beneficial to LMIC trainees (*N* = 65)				
Strongly disagree	–	–	8	12.3
Disagree	–	–	0	0.0
Neutral	–	–	0	0.0
Agree	–	–	13	20.0
Strongly agree	–	–	44	67.7
Trainee projects are relevant to the needs of the host country (U.S., *N* = 54; LMIC, *N* = 51, mentor only)				
Strongly disagree	2	3.7	2	3.9
Disagree	0	0.0	0	0.0
Neutral	7	13.0	4	7.8
Agree	29	53.7	20	39.2
Strongly agree	16	29.6	25	49.0
Trainees create a burden on the host institution (U.S., *N* = 66; LMIC, *N* = 65)				
Strongly disagree	25	37.9	25	38.5
Disagree	25	37.9	25	38.5
Neutral	6	9.1	6	9.2
Agree	7	10.6	7	10.8
Strongly agree	3	4.5	2	3.1
Trainees have a clear understanding of their expectations at the start of the fellowship (U.S., *N* = 50; LMIC, *N* = 51, mentor only)				
Strongly disagree	0	0.0	0	0.0
Disagree	2	4.0	4	7.8
Neutral	5	10.0	7	13.7
Agree	30	60.0	33	64.7
Strongly agree	13	26.0	7	13.7
Host institutions are provided with a clear description of the trainees’ level of training (U.S., *N* = 49; LMIC, *N* = 52, mentor only)				
Strongly disagree	0	0.0	0	0.0
Disagree	6	12.2	3	5.8
Neutral	8	16.3	12	23.1
Agree	24	49.0	28	53.8
Strongly agree	11	22.4	9	17.3
Trainees’ overall preparedness level at the beginning of their training year (U.S., *N* = 51; LMIC, *N* = 53, mentor only)				
Completely unprepared	1	2.0	3	5.7
Satisfactorily prepared	26	51.0	39	73.6
Well prepared	24	47.1	11	20.8

LMIC = low- and middle-income country; U.S. = United States.

**Figure 2.**
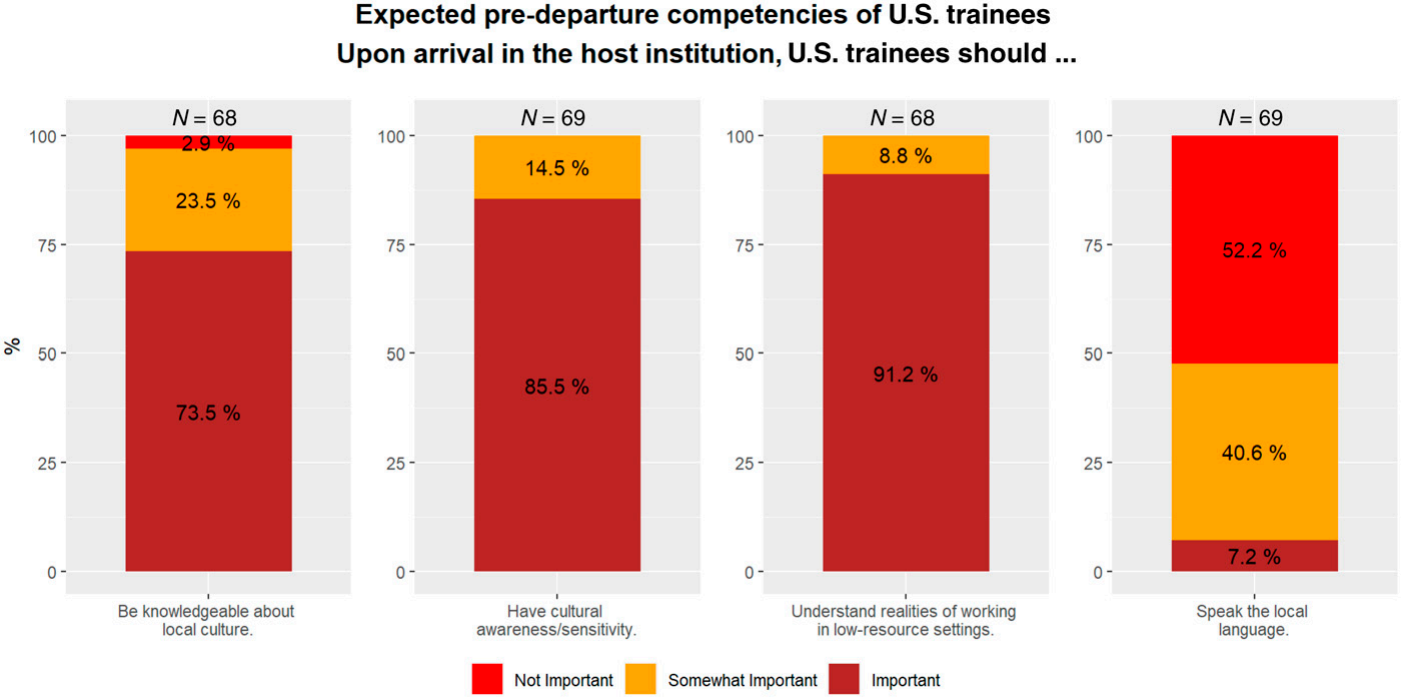
Desired cultural competencies of U.S. trainees from institutions hosting U.S. trainees.

Certain attributes were rated universally by respondents with a mentor role as being important or somewhat important for both U.S. and LMIC trainees to have upon arrival in the host country or institution (Table 5). These included demonstrating humility (U.S., 98%; LMIC, 94%), being confident (100% both the U.S. and LMIC), recognizing one’s personal limitations (U.S., 100%; LMIC, 98%), understanding health and human rights (100% both the U.S. and LMIC), and being knowledgeable about cultural perceptions of disease (U.S., 98%; LMIC, 100%). The majority of survey respondents from institutions hosting U.S. trainees rated it “important” that, upon arrival in the host country, U.S. trainees should be knowledgeable about the local culture (*n* = 50, 73.5%), have cultural awareness/sensitivity (*n* = 59, 85.5%), and understand the realities of working in low-resource settings (*n* = 62, 91.2%) (Figure 2). Only five respondents (7.2%) indicated speaking the local language was important.

**Table 5:** Desired personal and knowledge competencies of trainees from respondents with a mentor role

Variable	U.S. trainees		LMIC trainees	
	*n*	%	*n*	%
Demonstrate humility (U.S., *N* = 53; LMIC, *N* = 50)				
Not important	1	1.9	3	6.0
Somewhat important	9	17.0	12	24.0
Important	43	81.1	35	70.0
Be confident (U.S., *N* = 53; LMIC, *N* = 52)				
Not important	0	0.0	0	0.0
Somewhat important	21	39.6	20	38.5
Important	32	60.4	32	61.5
Recognize one’s personal limitations (U.S., *N* = 53; LMIC, *N* = 52)				
Not important	0	0.0	1	1.9
Somewhat important	10	18.9	9	17.3
Important	43	81.1	42	80.8
Demonstrate skill in evidence-based program planning and implementation (U.S., *N* = 53; LMIC, *N* = 51)				
Not important	1	1.9	1	2.0
Somewhat important	23	43.4	28	54.9
Important	29	54.7	22	43.1
Understand health and human rights (U.S., *N* = 53; LMIC, *N* = 52)				
Not important	0	0.0	0	0.0
Somewhat important	9	17.0	7	13.5
Important	44	83.0	45	86.5
Be knowledgeable about cultural perceptions of disease (U.S., *N* = 53; LMIC, *N* = 52)				
Not important	1	1.9	0	0.0
Somewhat important	17	32.1	13	25.0
Important	35	66.0	39	75.0
Understand cultural effects on patient behavior (U.S., *N* = 53; LMIC, *N* = 51)				
Not important	1	1.9	1	2.0
Somewhat important	14	26.4	13	25.5
Important	38	71.7	37	72.5

LMIC = low- and middle-income country; U.S. = United States.

In terms of professionalism (Table 6), most respondents in a mentor role agreed or strongly agreed that both U.S. and LMIC trainees demonstrated professional behavior (98% and 93%, respectively), were rarely late or unexpectedly absent (86% and 76%, respectively), listened actively (96% and 91%, respectively), and were receptive to constructive feedback (96% and 90%, respectively). Both U.S. and LMIC trainees were described as able to take initiative (94% and 79%, respectively), had the appropriate knowledge/skills to carry out their projects (91% and 76%, respectively), and met deadlines/timelines set for their projects (89% and 68%, respectively). Respondents also acknowledged that most trainees respected all members of the team (U.S., 96%; LMIC, 95%), maintained professional boundaries in their work relationships (U.S., 78%; LMIC, 80%), and modeled ethical behavior (U.S., 90%; LMIC, 91%) (Table 6). Both U.S. and LMIC trainees were described as not engaging in activities that undervalue or question mentor decision making (U.S., 85%; LMIC, 87%) or that may harm morale at the institution (U.S., 80%; LMIC, 84%) or reflect negatively on the institution or country (81%, both U.S. and LMIC).

**Table 6:** Professionalism, behavior, and knowledge of trainees from respondents with a mentor role

Trainees	U.S. trainees		LMIC trainees	
	*n*	%	*n*	%
Demonstrate professional behavior (U.S., *N* = 52; LMIC, *N* = 54)				
Strongly disagree	0	0.0	0	0.0
Disagree	0	0.0	0	0.0
Neutral	1	1.9	4	7.4
Agree	30	57.7	30	55.6
Strongly agree	21	40.4	19	35.2
Were rarely late or unexpectedly absent (U.S., *N* = 51; LMIC, *N* = 54)				
Strongly disagree	1	2.0	4	7.4
Disagree	1	2.0	0	0.0
Neutral	5	9.8	9	16.7
Agree	23	45.1	26	48.1
Strongly agree	21	41.2	15	27.8
Listened actively (U.S., *N* = 51; LMIC, *N* = 53)				
Strongly disagree	0	0.0	0	0.0
Disagree	0	0.0	0	0.0
Neutral	2	3.9	5	9.4
Agree	30	58.8	29	54.7
Strongly agree	19	37.3	19	35.8
Were receptive to constructive feedback (U.S., *N* = 51; LMIC, *N* = 53)				
Strongly disagree	0	0.0	0	0.0
Disagree	1	2.0	1	1.9
Neutral	1	2.0	4	7.5
Agree	31	60.8	32	60.4
Strongly agree	18	35.3	16	30.2
Took the initiative (U.S., *N* = 51; LMIC, *N* = 53)				
Strongly disagree	0	0.0	0	0.0
Disagree	0	0.0	1	1.9
Neutral	3	5.9	10	18.9
Agree	29	56.9	28	52.8
Strongly agree	19	37.2	14	26.4
Possess appropriate research knowledge and skills (U.S., *N* = 51; LMIC, *N* = 53)				
Strongly disagree	0	0.0	0	0.0
Disagree	1	2.0	2	3.8
Neutral	4	7.8	11	20.8
Agree	36	70.6	36	67.9
Strongly agree	10	19.6	4	7.5
Met project deadlines and timelines (U.S., *N* = 51; LMIC, *N* = 53)				
Strongly disagree	0	0.0	0	0.0
Disagree	1	2.0	3	5.7
Neutral	5	9.8	14	26.4
Agree	33	64.7	33	62.3
Strongly agree	12	23.5	3	5.7
Respected all team members (U.S., *N* = 51; LMIC, *N* = 53)				
Strongly disagree	0	0.0	0	0.0
Disagree	0	0.0	0	0.0
Neutral	2	3.9	3	5.7
Agree	27	52.9	28	52.8
Strongly agree	22	43.1	22	41.5
Maintained professional boundaries (U.S., *N* = 51; LMIC, *N* = 54)				
Strongly disagree	1	2.0	1	1.9
Disagree	1	2.0	0	0.0
Neutral	9	17.6	10	18.5
Agree	26	51.0	29	53.7
Strongly agree	14	27.5	14	25.9
Modeled ethical behavior (U.S., *N* = 51; LMIC, *N* = 54)				
Strongly disagree	0	0.0	0	0.0
Disagree	0	0.0	0	0.0
Neutral	5	9.8	5	9.3
Agree	32	62.7	34	63.0
Strongly agree	14	27.5	15	27.8
Engaged in activities that undervalue or question mentor decision making (U.S., *N* = 51; LMIC, *N* = 54)				
Strongly disagree	12	23.5	19	35.2
Disagree	31	60.8	28	51.9
Neutral	3	5.9	3	5.6
Agree	3	5.9	4	7.4
Strongly agree	2	3.9	0	0.0
Engaged in activities that harm morale (U.S., *N* = 51; LMIC, *N* = 54)				
Strongly disagree	20	39.2	22	40.7
Disagree	21	41.2	23	42.6
Neutral	5	9.8	4	7.4
Agree	2	3.9	2	3.7
Strongly agree	3	5.9	3	5.6
Engaged in activities that reflect negatively on the institution or country (U.S., *N* = 52; LMIC, *N* = 54)				
Strongly disagree	17	32.7	19	35.2
Disagree	25	48.1	25	46.3
Neutral	9	17.3	8	14.8
Agree	1	1.9	2	3.7
Strongly agree	0	0.0	0	0.0

LMIC = low- and middle-income country; U.S. = United States.

**Figure 3.**
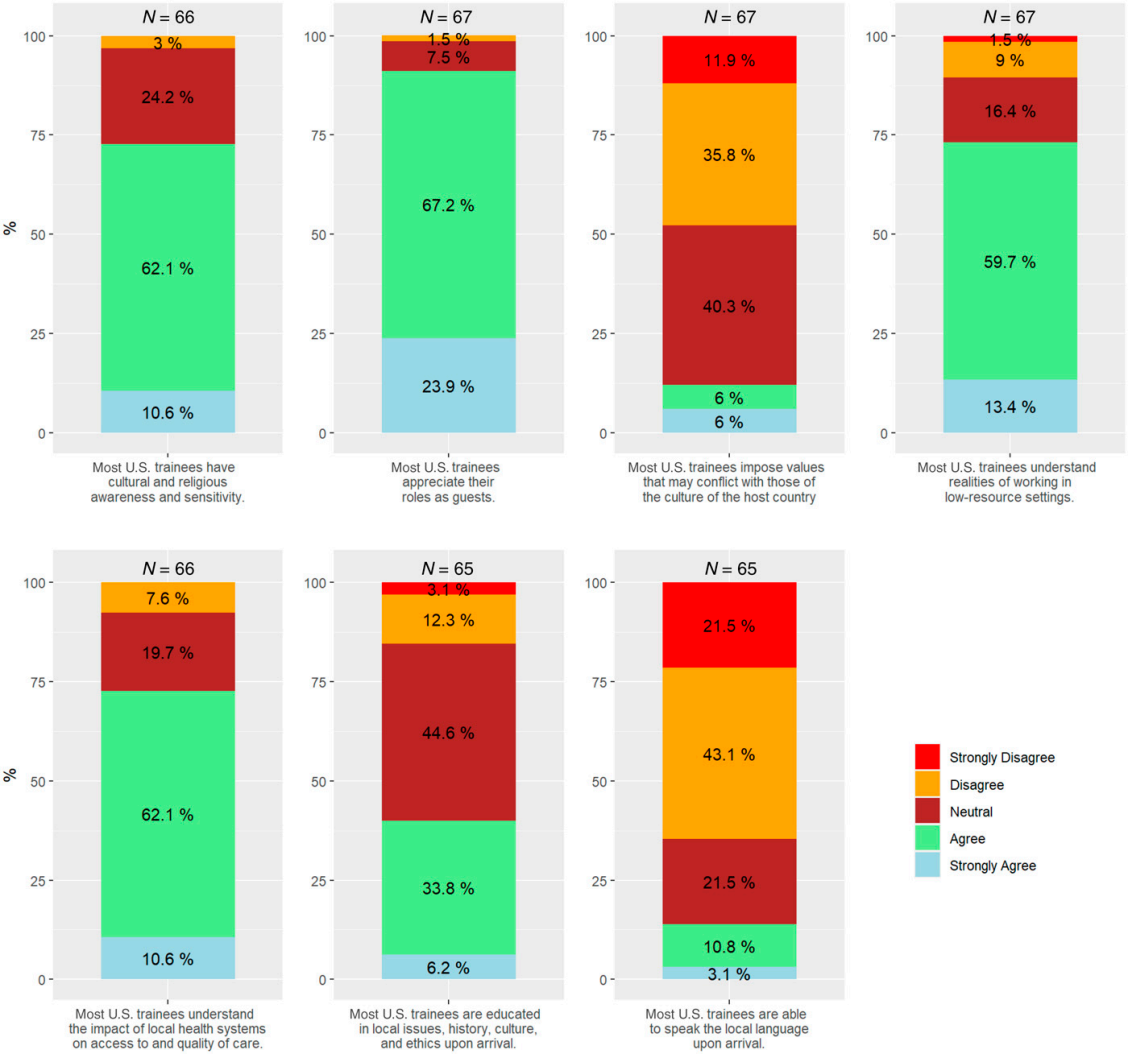
Observed cultural competencies of U.S. trainees from institutions hosting U.S. trainees.

Reflecting on observed cultural competencies of U.S. trainees, 73% of respondents agreed that most U.S. trainees have cultural and religious awareness and sensitivity, and most appreciated their roles as guests (91.1%) (Figure 3). Only 12% of respondents agreed or strongly agreed that U.S. trainees tended to impose values that may conflict with those of the host country’s culture. Sixty-five percent of respondents acknowledged that most U.S. trainees were not able to speak the local language upon arrival (64.6%) (Figure 3).

### Qualitative survey results.

Respondents provided additional comments to support or clarify their responses on 20 of the survey items. Three main themes emerged from these data: 1) enhancing local program ownership and LMIC trainee experiences, 2) prioritizing preparation for host sites and U.S. trainees, and 3) proposing future directions. Detailed descriptions of these themes are provided next, with themes mapped to key quantitative outcomes in Table 3.

#### Enhancing local program ownership and LMIC trainee experiences.

Program mentors and administrators from LMIC host institutions expressed a desire to have a more prominent role in the management of the FGHFS program at their respective sites. Drawing from previous experiences as participating sites, several respondents cautioned that lack of host site involvement in the trainee selection process could result in trainees whose projects are not aligned with existing projects or priorities within their institution. The provision of additional funding support, institutional capacity-building mechanisms, and a more active role in programmatic decision-making processes were perceived as ways of increasing the ability of sites to facilitate the program effectively.

Respondents provided numerous ways through which the FGHFS program could be improved to enhance LMIC trainee experiences, including increasing available research funds, offering program extensions, facilitating bidirectional exchanges, and offering additional ways for U.S. and LMIC trainees to engage in research collaborations. The annual orientation all trainees receive at the NIH campus at the beginning of their fellowships was identified as a critical period for expectation setting for both trainees and their mentors. A few individuals stated expressly that the orientation should be offered in person again (because it was held virtually during the COVID-19 pandemic) to communicate program requirements more effectively and allow networking opportunities for LMIC trainees.

#### Prioritizing preparation for host sites and U.S. trainees.

Respondents identified aspects of programmatic support in the program preparation phase that could be provided or strengthened to support effectively those trainees hosted at their institutions, including obtaining support for the program from institutional leaders. Individuals also requested guidance on how to select and prepare mentors, reiterating that early involvement of and communication with the host sites help reduce problems after trainees arrive.

Respondents also described how prioritizing prearrival U.S. trainee engagement is important for overall program success. All but one respondent, who provided a comment regarding prearrival competencies for U.S. trainees, stated that language training, if necessary, and advanced research skills or content knowledge can be learned in-country via the mentored research experience and local trainings. As such, individuals felt the program should instead prioritize efforts to make U.S. trainees aware of the research setting realities at their host sites. This can be facilitated by early communication between trainees and LMIC mentors prior to deployment to the LMIC sites, and by encouraging additional capacity-building activities if trainees have time outside of their proposed projects.

Safety of trainees was mentioned across responses to several different question prompts, each time emphasizing that trainees need to have an awareness of cultural norms and local laws. Most respondents shared the sentiment that U.S. trainees are respectful and “they are [culturally] sensitive, but not very [culturally] aware” (no. 53). Individuals felt host institutions were well-positioned to offer virtual pretravel sessions to provide U.S. trainees with general knowledge on the health-care system, institutional policies, and research ethics requirements, and to increase their awareness of relevant, local sociocultural issues.

#### Proposing future directions.

Respondents identified several ways in which the program could grow to support long-term goals of increasing their institution’s reputation for research eminence. Several respondents suggested adding a local research dissemination requirement. Dissemination outputs were described as ways to ensure trainees meet their research objectives, “inspire other trainees and build capacity” (no. 69), make knowledge accessible that could “benefit other LMICs” (no. 19), and strengthen collaborations with other institutions. However, to support trainee research dissemination efforts, these individuals also recognized that sites would need to provide training and ongoing mentorship on manuscript development, and that the program would need to allow budgeting for dissemination costs, such as publication fees and postfellowship conference attendance.

To advance trainee and institutional visibility, respondents identified program support structure gaps that need to be addressed in future program iterations. One individual requested that FGHFS consortium leaders provide the sites with feedback; another highlighted the need to receive trainee feedback on program quality. Some individuals felt trainees should have to provide regular progress updates to ensure project deliverables are produced and can be used to demonstrate visibly the presence of the program at their institutions. As summarized by one respondent, there was a stated desire across responses to “create more opportunities for networking among fellows [and] engage more actively with administrators of the host institution to create more visibility for the program and more recognition for the trainee as well as to attract bigger talent” (no. 79).

### Perception of program needs based on number of trainees hosted.

Comparisons of individual responses by the number of trainees an institution hosted (threshold of 11 fellows) identified differences in perceptions of how the program benefits host institutions, with a stronger emphasis on capacity building for staff (Supplemental Table 1). There was mixed consensus regarding how to support protected time for trainees and limit burdens to host institutions, with sites that have hosted fewer than 11 fellows advocating for mechanisms to allow fellows to focus on their research project, including salary coverage. However, individuals from sites who hosted 11 or more fellows felt salary coverage and program expectations were clear. Suggested methods for limiting burdens to the host institutions, unique to each level of program experience, included covering the cost of research equipment (< 5 fellows), having paid administrative staff (≥ 11 fellows), and focusing on advanced preparation (5–10 fellows). Most felt salaries for mentors should be included and sites should have access to additional resources from the U.S. institutions. Regardless of the host institution’s experience with the program, all had positive impressions of the program and felt trainee advancement would be best supported by increased opportunities to network and partner with U.S. fellows (Supplemental Table 1).

## DISCUSSION

We present multidomain perspectives covering a range of LMIC personnel from 31 mainly African and Asian countries involved in oversight or mentorship of U.S. and LMIC trainees at LMIC host institutions. Respondents were generally involved with the program for about 8 years, and spent between 6 and 20 contact hours per month with each trainee. This survey not only elucidated the adaptive attributes of U.S. trainees hosted in LMIC institutions, but also described the positive impact of U.S. and LMIC trainees on a spectrum of themes related to needs and expectations of host institutions. In addition, we highlighted the desire of LMIC host institutions to be engaged in program decision making, including selection of potential mentees and autonomy in grant management.

Specifically, we showed some concordance of perception that host institutions received enough funds to support trainees. Respondents also acknowledged that their institutions have, over time, acquired the capacity to manage subcontracts and handle funds associated with the program. This finding is not entirely unexpected, as U.S. and LMIC institutions have partnered to invest heavily in capacity building for skills acquisition, ranging from grant writing to grant management for LMIC partner institutions. This investment has yielded positive outcomes, with LMIC host institutions becoming more confident in their ability to manage resources locally.

Importantly, we found evidence of trainees maintaining long-term contact with their LMIC host institutions through collaborative projects, papers, and grants. Building successful and equitable programs with lasting impact will require such long-term partnerships. There is, however, room for improvement in areas of feedback to and from trainees, site administrators, and leaders.

Overall, FGHFS fostered a positive impression of LMIC and U.S. trainees within its LMIC host institutions. Respondents found the program beneficial to the host institutions and to LMIC trainees, and relevant to the needs of the host country. We also gained greater insight into expectations that both U.S. and LMIC trainees should exhibit attributes such as demonstrating humility, being confident, recognizing one’s personal limitations, understanding health and human rights, and being knowledgeable about cultural perceptions of disease. Other desirable qualities included understanding cultural effects on patient behavior, acknowledging patient barriers to accessing health care, and appreciating the role of local public health systems. Our findings are consistent with a recent mixed-methods study^22^ of global health clinical trainees and their mentors that highlighted the importance of visiting trainees possessing key personal attributes of humility, respect, and flexibility.

We found that both U.S. and LMIC trainees demonstrated professional behavior, in terms of punctuality, general habits, and attitudes; and were described as being courteous, displaying initiative, and working within the limits of their knowledge and skills. This finding is reassuring, because performing outside the scope of practice is a recognized ethical risk among health-care professionals and trainees working in LMICs.^23^

The unique cultural heritage of persons living in LMICs binds their shared experience and serves as a manifest to their views on common global social etiquette and practices. One of the factors that fosters respectful and productive working relationships is understanding local customs and sensitivities. Although the ability of trainees to speak local languages was listed as a positive attribute, the lack of this skill set did not appear to impede U.S. trainees from carrying out their activities effectively. English fluency and availability of interpreters at some host institutions may be responsible for this finding.

Respondents to our survey made important suggestions that can be adopted to improve the program. These include enhancing the research dissemination requirement to make new knowledge accessible that could benefit other LMIC countries and strengthen collaborations with other institutions. Research dissemination via peer-reviewed publications is a cardinal objective of the program, but other avenues for dissemination to local partners and stakeholders can be emphasized further as well. To support trainee dissemination efforts, respondents suggested the program should provide training and ongoing mentorship on manuscript development, and support expenses for dissemination costs, such as publication fees and conference attendance postfellowship. This recommendation is logical, because the postfellowship period is when most trainees have sufficient results to present. The dissemination of findings to local audiences will also help narrow the research-to-policy gap, which tends to be pronounced in LMIC settings, in part because of disparities in access to scientific publications in such settings.^24^

Respondents also suggested that LMIC trainees would benefit from opportunities to work in the U.S. mentor’s laboratory or facility to gain exposure to different research settings. In fact, FIC added a requirement for this component in the Request for Applications, issued in 2021, for the next 5-year cycle of the program—TW-21-004: Launching Future Leaders in Global Health (LAUNCH) Research Training Program.

Last, effective global health capacity development requires a framework of mutual respect, transparent communication, and intentional engagement of all parties at the decision-making level. We need equitable partnerships that create prospects to equalize access and opportunities of educational experiences.^25^ Periodic evaluation of such partnerships using objective benchmarks and indicators will enhance accountability, improve program performance, and promote the development of sustainable initiatives that are responsive to local needs and anchored on shared objectives.

## Supplemental files


Supplemental materials
