# Supplementary material for: LMIC Research Centers’ Experiences Hosting U.S. and LMIC Trainees: Evaluation of the Fogarty Global Health Fellows and Scholars Program, 2012 to 2020
Source: Am J Trop Med Hyg. 2023 Feb 20;108(4):660–71. doi: 10.4269/ajtmh.22-0595 (PMC10076995; doi:10.4269/ajtmh.22-0595)
Supplement: Supplementary file 1 [file tpmd220595.SD1.pdf]

Supplementary Material

Table S1: Summary of respondents' views according to the number of fellows hosted by their institutions

	Number of Fellows Hosted		
	**< 5**	**5-10**	**11+**
**Ways to enhance benefits to host institution**	Strong* emphasis on capacity building for staff and increase in program capacity to accommodate more fellows.	Strong emphasis on strengthening networks and collaborations between host and U.S. institutions.	Strong emphasis on capacity building for staff and access to resources to support fellowship activities.
**How to support trainee advancement**	Strong emphasis on support for dissemination activities, networking with other fellows, adequate research planning and supportive supervision.	Emphasis on bi-directional exchange activities to provide fellows from LMICs opportunities to visit U.S. institutions to acquire relevant skills.	Strong emphasis on U.S./LMIC fellow partnerships to conduct collaborative research.
**How to support protected time for trainees**	Strong emphasis on salary coverage for the fellows, having no or limited competing projects, adequate research planning, having leadership buy-in, and access to resources.	Strong emphasis on salary coverage and supportive supervision to make sure that fellows focus on their projects.	Strong emphasis on salary coverage and a focus on advance preparation so that trainees know exactly what they are expected to do.
**Ways to limit burdens to host institution**	Strong emphasis on expanding research funding to cover cost of buying equipment, access to resources from U.S. institutions, as well as salary coverage for both mentors and trainees.	Emphasis on having paid administrative staff to support fellows, and salary coverage for both mentors and trainees.	Strong emphasis on having access to resources from U.S. institutions, and a focus on advance preparation to enable both the host institutions and fellows know what is expected from them.
**Host institution impressions about trainees or program**	Generally good impressions. Negative impressions directed at specific trainee/ fellow and not the program.	Generally good impressions.	Generally good impressions. Negative impressions directed at specific trainee/ fellow and not the program.
**Host institution impressions about U.S. trainees**	Culturally sensitive and respectful.	Culturally sensitive and respectful.	Culturally sensitive.

*Strong emphasis = having at least 3 mentions; U.S. = United States; LMICs = low- and middle-income countries
